# The First Gynandromorph of Stonefly From China (Plecoptera: Leuctridae)

**DOI:** 10.1002/ece3.72378

**Published:** 2025-10-24

**Authors:** Xiao Yang, Qing‐Bo Huo, Yu‐Zhou Du

**Affiliations:** ^1^ College of Plant Protection & Institute of Applied Entomology Yangzhou University Yangzhou China; ^2^ Jiangsu Province Engineering Research Center of Green Pesticides Yangzhou University Yangzhou China

**Keywords:** China, gynandromorph, *Paraleuctra cercia*, Plecoptera

## Abstract

The distribution of *Paraleuctra cercia* (Okamoto, 1922) in China is confirmed based on a recent collection from Heilongjiang Province, northeastern China. Meanwhile, a gynandromorph of *P. cercia* is discovered among the specimens, as the first report of a gynandromorph stonefly from China with high‐definition color images provided.

## Graphical abstract

The distribution of *Paraleuctra cercia* (Okamoto, 1922) in China is confirmed. A gynandromorph of *P. cercia* is discovered as the first report of a stonefly species for the country.

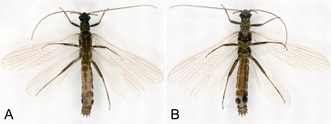

## 1 Introduction

*Paraleuctra cercia* (Okamoto 1922) is a common species of the family Leuctridae in Northeast Asia, widely distributed in Russia and Korea. Tang et al. (2024) claimed to have found this species in Northeast China and regarded it as a new country record, but they only noted the collected information in their paper without providing any descriptions or figures. Recently, we examined a collection of Leuctridae from Heilongjiang Province, northeastern China, and confirmed the specimens as *Paraleuctra cercia* through morphological identification. Our study further confirms the distribution of this species in China by providing straight pictorial evidence of both male and female adults.

In the specimens of *P. cercia*, we discovered a gynandromorph. This individual exhibits a female subgenital plate with male epiproct and paraproct together. Previously, the gynandromorph of insects seemed not to be so rare, but it was seldom reported in Plecoptera, and only recorded in a few Euholognatha families (Notonemouridae, Capniidae, Nemouridae, and Leuctridae) (Hanada and Yamamoto 2023). In the genus *Paraleuctra* of Leuctridae, the gynandromorph specimen of only *P. occidentalis* (Banks 1907) has been reported (Ricker 1965). Here, we describe this male and female heterotypic specimen in detail and compare it with normal male and female adults. This study presents the second case of gynandromorph in *Paraleuctra* species and also represents the first such report in the stonefly of China.

## 2 Material and Methods

Specimens were collected by hand and preserved in 75% ethanol. Morphological details were examined with a Leica MZAPO microscope. Color illustrations were taken with a KEYENCE VHX‐5000. All specimens used in this study are deposited in the Insect Collection of Yangzhou University (ICYZU), Jiangsu Province, China.

## 3 Result

### 3.1 *Paraleuctra cercia* (Okamoto, 1922)

*Leuctra cercia* Okamoto, 1922, 37.

*Leuctra forficularis* Okamoto, 1922, 39.

*Leuctra higashiyamae* Kohno, 1964, 33.

*Paraleuctra cercia*: Illies, 1966, 113.

*Paraleuctra cercia*: Cao et al., 2021, 1.

#### 3.1.1 Materials Examined

Three males, four females, one gynandromorph, China, Heilongjiang Province, Tahe county, Huma River Bridge, 52.304958 N, 124.696754 E, 2023‐VI‐6~7, leg. Ya‐Fei Zhu, Xiao Yang.

#### 3.1.2 Description

**FIGURE 1:**
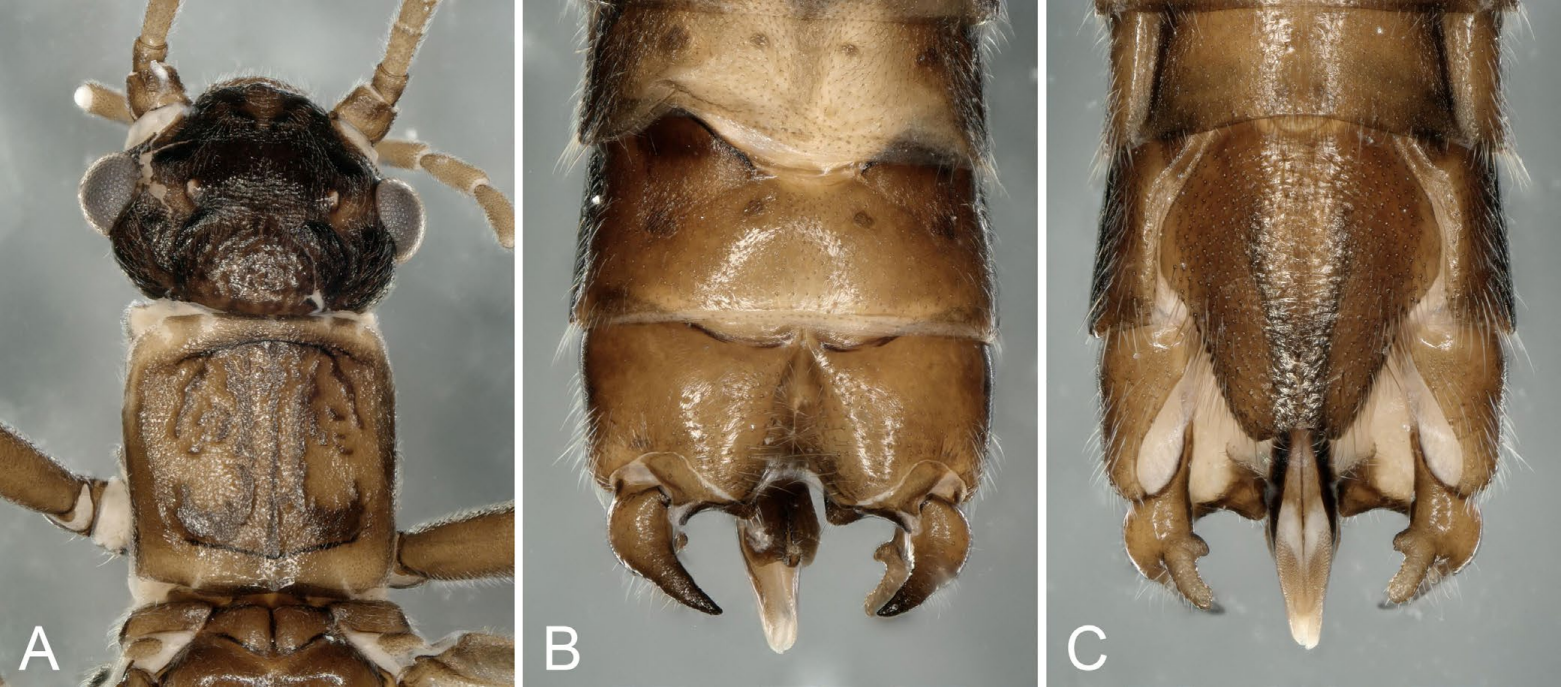
Male of *Paraleuctra cercia*: (A) head and pronotum, dorsal; (B) terminalia, dorsal; (C) terminalia, ventral.

**Male:** Body length 5.0–7.7 mm. Cerci strongly swollen at the base (Figure 3), less curved, the tips of the teeth directed backwards; ventral tooth with an additional tooth at the base, dorsal tooth without it (Figure 1).

**FIGURE 2:**
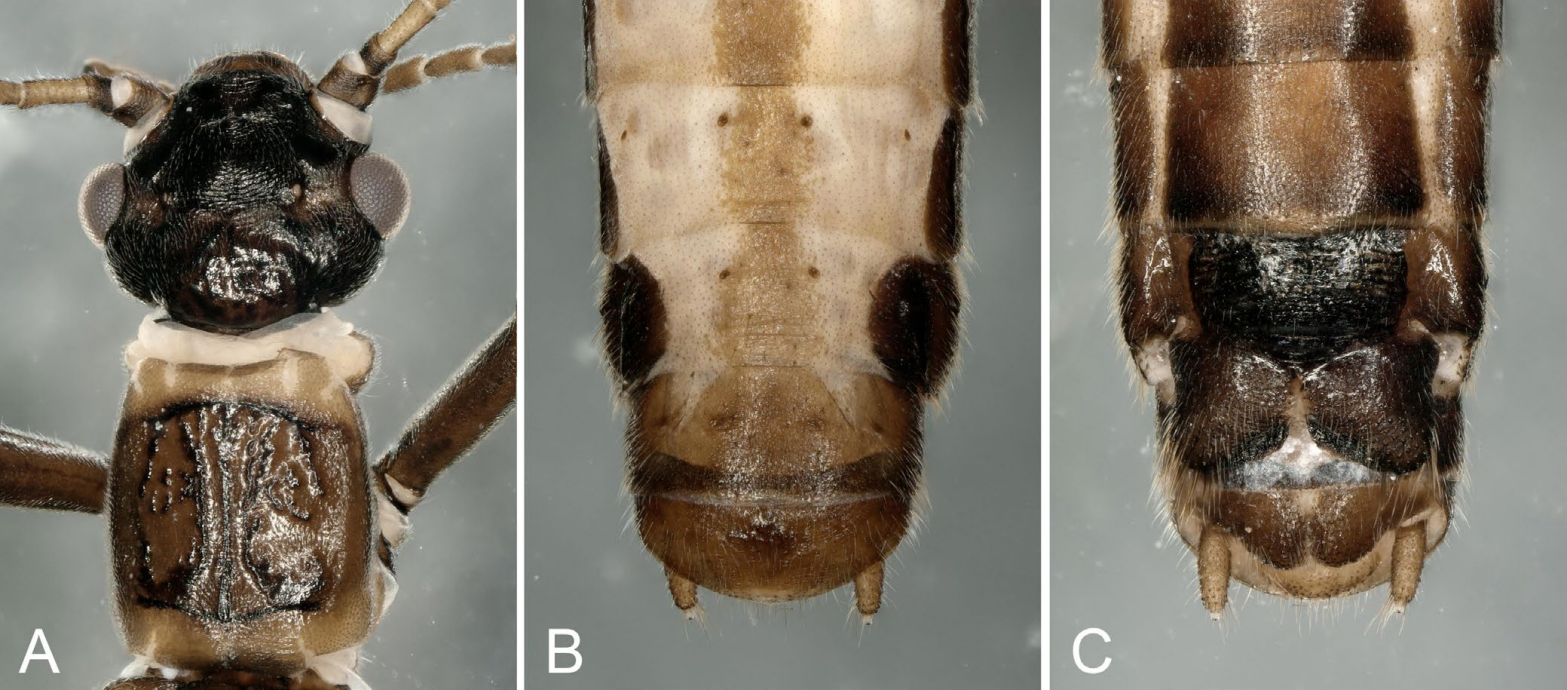
Female of *Paraleuctra cercia*: (A) head and pronotum, dorsal; (B) terminalia, dorsal; (C) terminalia, ventral.

**Female:** Body length 6.0–9.0 mm. The lateral edges of the lobes of the subgenital plate are parallel, with the basal part narrower, separated from the wide distal part by a sharp ledge (Figure 2).

The features of male and female adults of this species are shown in Figures 1 and 2. The identification is according to Teslenko and Zhiltzova (2009).

**FIGURE 3:**
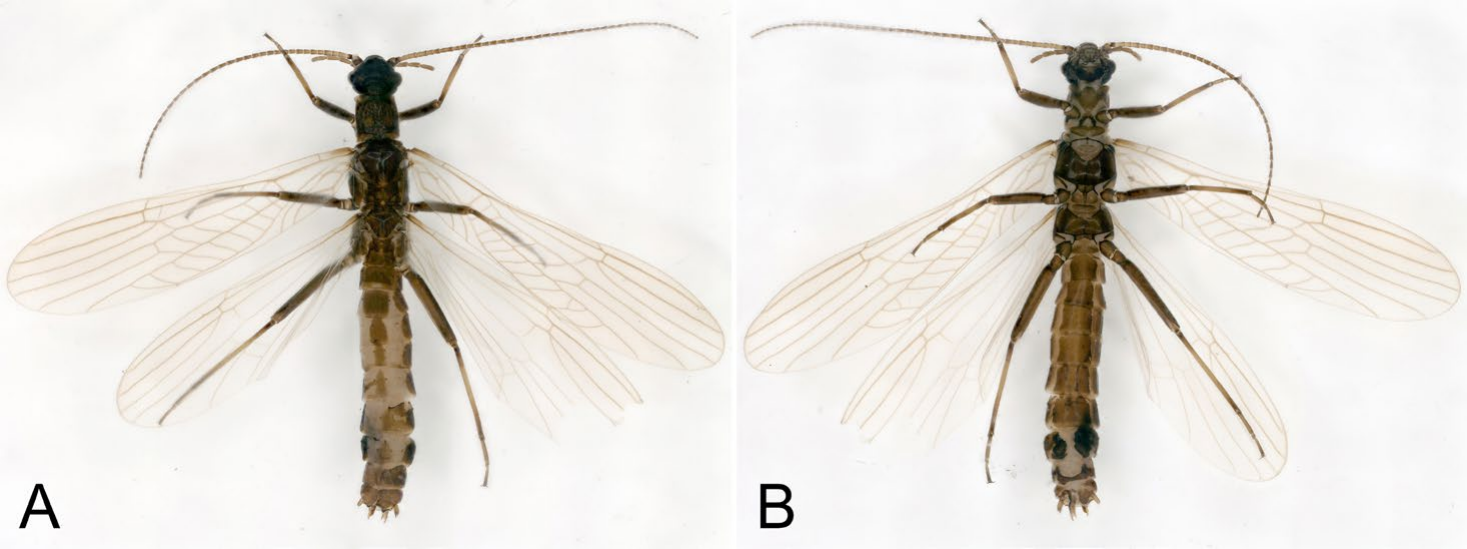
Gynandromorph specimen of *Paraleuctra cercia*, habitat: (A) dorsal; (B) ventral.

**Gynandromorph:** The left and right female and male characteristics of this gynandromorph are produced in a mosaic pattern, and the left and right male and female characteristics are not completely separated (Figure 3). As is the case with most stonefly gynandromorphs, the differences in the abdomen were most striking.

**FIGURE 4:**
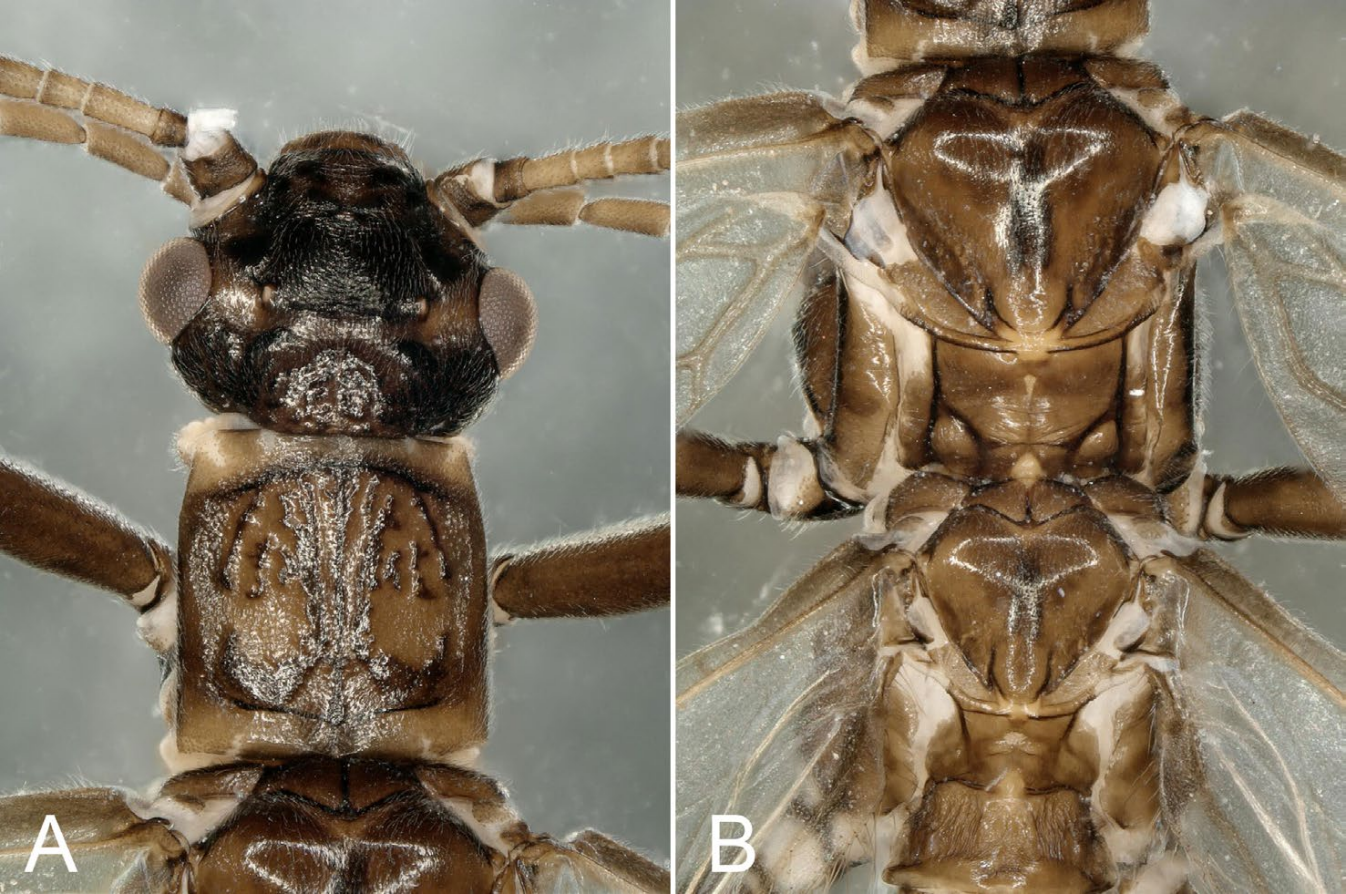
Gynandromorph specimen of *Paraleuctra cercia*: (A) head and pronotum, dorsal; (B) mesothorax and metathorax, dorsal.

**FIGURE 5:**
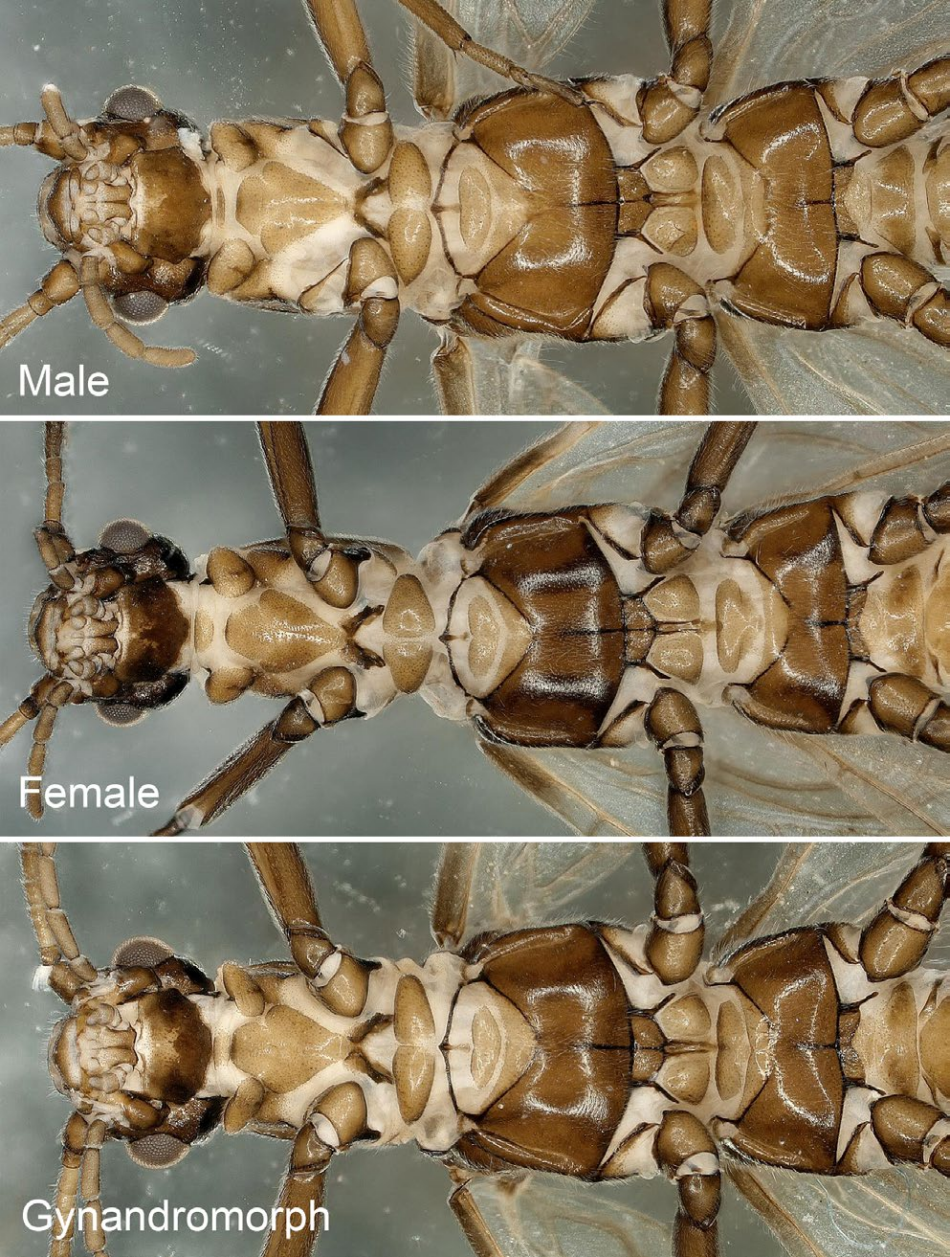
Head and thoraxes of *Paraleuctra cercia*, ventral.

In this study, we carefully compared the head, thoraxes (dorsal and ventral) (Figures 4 and 5), wings, and feet of this individual, and confirmed that its upper body did not present any cross‐gender mosaics: there is no visible asymmetry or “splicing” along (Figures 4 and 5).

**FIGURE 6:**
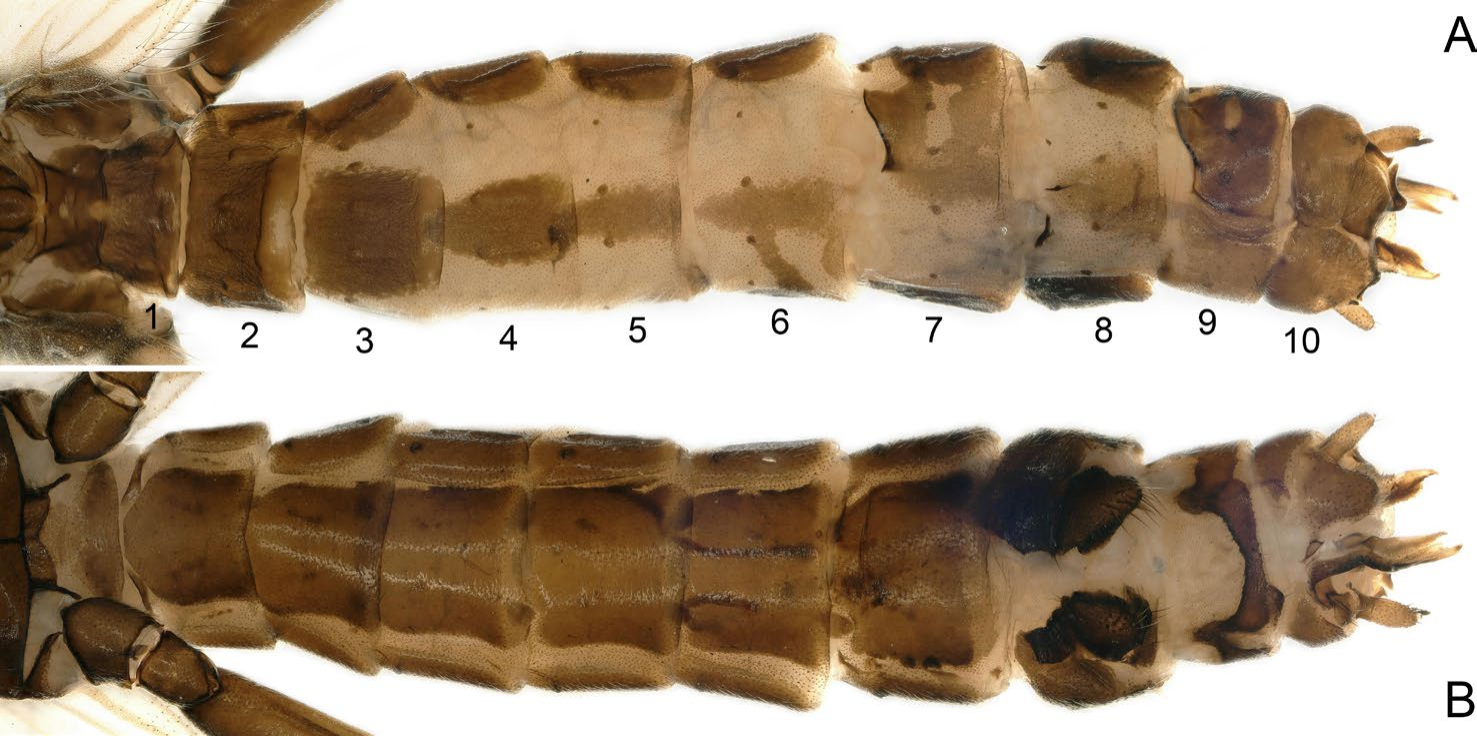
Gynandromorph specimen of *Paraleuctra cercia*, abdominal segments: (A) dorsal; (B) ventral.

**FIGURE 7:**
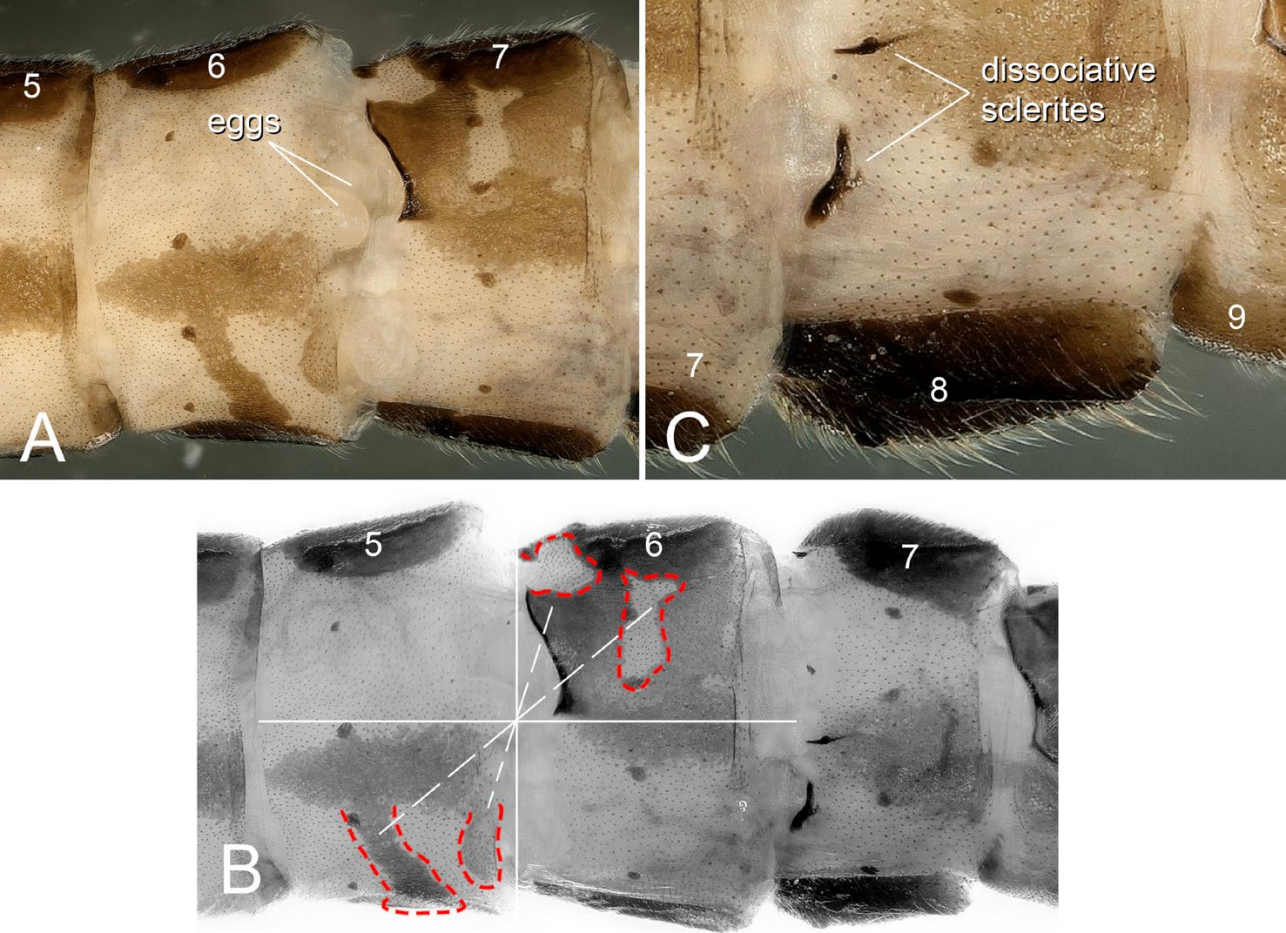
Gynandromorph of *Paraleuctra cercia*, abdominal segments: (A) eggs occurred between terga 6 and 7; (B) pattern of partial pigmentation on the left side (female) of tergum 5 and the right side (male) of tergum 6; (C): Dissociative sclerites at the left of tergum 8.

**FIGURE 8:**
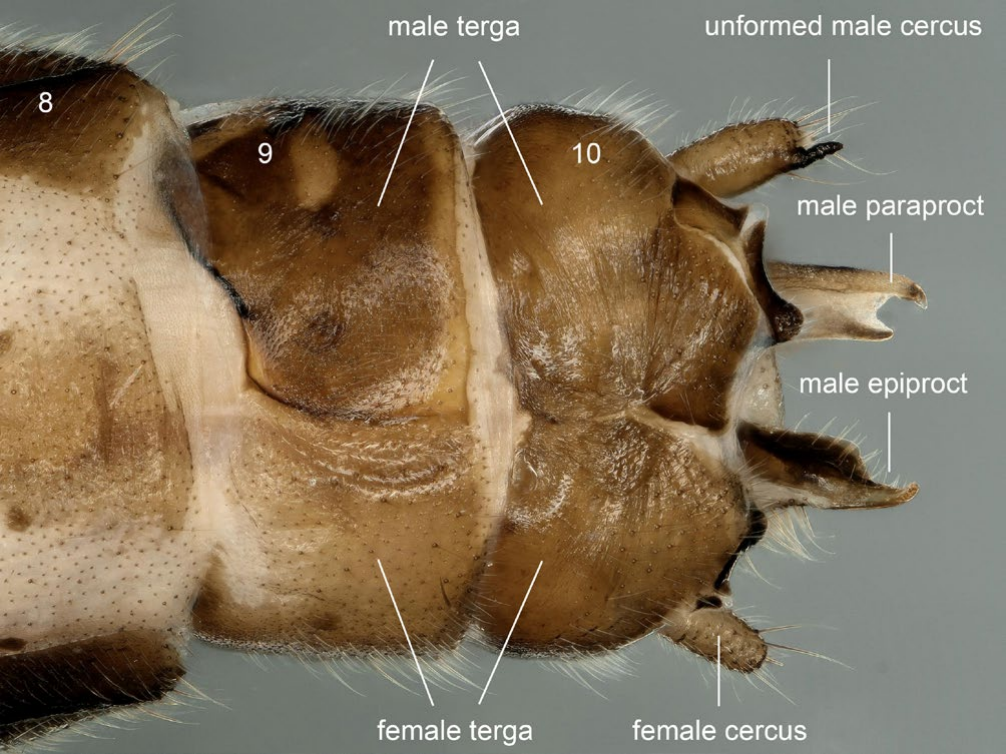
Gynandromorph specimen of *Paraleuctra cercia*, terminalia, dorsal.

In dorsal view, terga 1–4 unmodified. Sclerosis is evident on the right side of terga 5 and 7, absent on the left (Figure 6). Dissociated sclerites are present on the left side of tergum 8; ellipsoidal unfertilized eggs are observed at the junction of ventral segments 6 and 7. Most notably, partial pigmentation on the left (female) side of tergum 5 and the right (male) side of tergum 6 is arranged in central symmetry along the terga 5–6 dividing axis (Figure 7). The apex of the right cercus on tergum 10 is specialized, forming a small apical spine; the left cercus is unspecialized. The right side of tergum 10 is terminating in a hook‐like, dorsally curved supranal process (Figure 8). Ventral view: a small vesicle‐like mass is present at the terminus of segment 7 (Figures 6 and 7).

**FIGURE 9:**
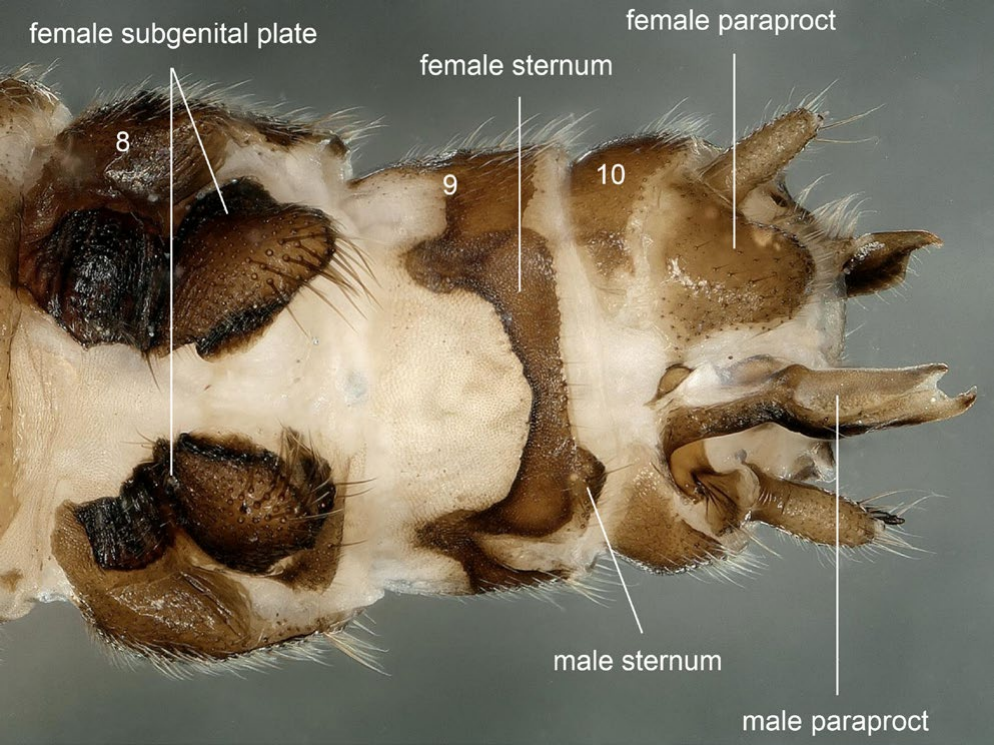
Gynandromorph specimen of *Paraleuctra cercia*, terminalia, ventral.

Sternum 7 with basally divided female subgenital plate, terminal rectangular with few long hairs; genital pore not discernible (Figure 9). Forked male paraproct on right side, and the triangular female paraproct on left side (Figure 9).

**Distribution:** China (Heilongjiang); Japan; Korea; Russia.

## 4 Discussion

Gynandromorphs are often noticed in the dimorphic or polymorphic insects (e.g., beetles, bees, ants, and butterflies) because the morphology or color pattern between the different sexes is dramatically different and visible (Joshua et al. 2015; Matsumoto and Knell 2017; Krichilsky et al. 2020). Commonly, gynandromorphs may be bilateral (divided down the middle into male and female sides, horizontally or vertically; axisymmetric or not), or mosaic with patches characteristic of one sex appearing in a body part characteristic of the other sex (Fusco and Minelli 2023).

However, the gynandromorphs may be overlooked in some species like stoneflies in which sexual dimorphism is less pronounced. Recently, Fusco and Minelli (2023) provide four schematic patterns of the most common symmetric gynandromorphism (Figure 9). But according to related studies in stoneflies, it seems that some structures may be partly reversed in a centrally symmetric manner, but all the male and female features are not arranged in a strictly horizontal or vertical symmetrical pattern. Therefore, the “mosaic limited to the abdominal terminalia” may be more applicable to other insects that lack sexual dimorphism.

Previous taxonomic classifications of insect sexual mosaicism recognized three primary categories: true gynandromorphism, intersexuality, and external genitalia teratology (Cui and Cai 2003; Soldán and Landa 1981; Wu et al. 1993). In model organisms like *Drosophila*, mosaic adults may display bilateral sexual differentiation along vertical/horizontal axes, though irregular mosaic patterns predominate. The spatial segregation of male/female cells was once considered to result from the random direction of the initial zygote division (Soldán and Landa 1981; Hotta and Benzer 1972). Biological evidence confirms that sexual mosaics may retain partial capacity for mating and reproduction: in *Habrobracon* wasps, gynandromorphs exhibit sex‐specific behavioral patterns contingent on cephalic morphology: specimens with male cephalic structures demonstrate courtship displays and copulatory attempts, while those with female‐dominant cephalic features display complete oviposition behavior (Clark and Egen 1975). This behavioral dichotomy is further quantified in *Drosophila* models. Through systematic analysis of 208 gynandromorphic flies, Hotta and Benzer (1972) documented: 130 individuals performed species‐typical wing vibration toward conspecific females (courtship initiation); 99 specimens engaged in mounting behavior (copulatory attempts); 23 cases achieved complete genital coupling (successful mating).

As the first gynandromorph stonefly reported from China, this specimen belongs to external genitalia teratology. In Plecoptera, male‐specific structures, particularly the epiproct (functionally analogous to a penis), remain vestigial or malformed in these gynandromorphs, contrasting sharply with the completeness of female morphological features (including the sclerotic subgenital plate and multiple visible eggs). This developmental asymmetry suggests severely compromised male mating functionality in gynandromorphic individuals. However, when encountering mate‐searching males (which initiate courtship via substrate‐borne vibrational signals and random tactile interactions) (Tierno de Figueroa et al. 2019; Huo et al. 2022), the gynandromorph may nevertheless maintain female receptivity and oviposition capacity. Notwithstanding these uncertainties, the reproductive plasticity of Plecoptera is underscored by this phenomenon, particularly when considered alongside their documented capacity for viviparity and parthenogenesis (Degrange 1958; Teslenko and Yavorskaya 2020). From a biogeographical perspective, our findings, along with previous studies from other countries, indicate that gynandromorphs occur in both Northern and Southern Hemisphere stoneflies (limited to Euholognatha members) without significant geographical constraints, appearing to be a widespread global phenomenon. However, gynandromorphs remain rare in all field surveys and have never been found in large numbers within the same collection of stoneflies. Therefore, we suggest that the sporadic nature of these cases implies that they are unlikely to be population‐level events caused by broad‐scale abiotic factors. Instead, they are more likely individual anomalies resulting from reproductive system abnormalities or microbial infections (Vance 1996). It is worth noting that researchers studying mayflies (order Ephemeroptera) hold a different view: they tend to attribute the occurrence of gynandromorphs to environmental factors such as temperature (Yang et al. 2023; Li et al. 2025). The mechanisms underlying such phenomena remain under debate, and further evidence is needed for in‐depth study.

## Author Contributions

**Xiao Yang:** conceptualization (equal), investigation (equal), visualization (equal), writing – original draft (equal). **Qing‐Bo Huo:** conceptualization (equal), visualization (equal), writing – original draft (equal). **Yu‐Zhou Du:** funding acquisition (equal), project administration (equal), writing – review and editing (equal).

## Conflicts of Interest

The authors declare no conflicts of interest.

## Data Availability

All data are available in this paper.
